# On Existence and Admissibility of Singular Solutions for Systems of Conservation Laws

**DOI:** 10.1007/s40819-022-01368-4

**Published:** 2022-06-28

**Authors:** Henrik Kalisch, Darko Mitrovic

**Affiliations:** 1 Department of Mathematics, University of Bergen, Faculty of Mathematics, Allegaten 41, 5007 Bergen, Norway; 2 Faculty of Mathematics, University of Vienna, Oskar Morgenstern-Platz 1, 1090 Vienna, Austria

**Keywords:** Conservation laws, Cauchy problem, Singular solutions, Uniqueness, Weak asymptotics, 35L65, 35L67, 35Q35

## Abstract

A weak notion of solution for systems of conservation laws in one dimension is put forward. In the framework introduced here, it can be shown that the Cauchy problem for any $n\times n$ system of conservation laws has a solution. The solution concept is an extension of the notion of singular $\delta$-shocks which have been used to provide solutions for Riemann problems in various systems, for example in cases where strict hyperbolicity or the genuine-nonlinearity condition are not satisfied, or in cases where initial conditions have large variation. We also introduce admissibility conditions which eliminate a wide range of unreasonable solutions. Finally, we provide an example from the shallow water system which justifies introduction of $\delta$-distributions as a part of solutions to systems of conservation laws.

## Introduction

We are interested in the general $n\times n$ system of one-dimensional conservation laws

1.1 $$\begin{aligned} {\left\{ \begin{array}{ll} &{}\partial _t u_1+ \partial _x f_1(\mathbf{u})=0,\\ &{} \qquad \vdots \\ &{}\partial _t u_d+ \partial _x f_d(\mathbf{u})=0, \end{array}\right. } \end{aligned}$$

where $\mathbf{u}=(u_1,\dots ,u_d)$. The system is augmented with the initial data

1.2 $$\begin{aligned} \mathbf{u}|_{t=0}=(u_1,\dots ,u_d)\Big |_{t=0}=(u_{10},\dots ,u_{d0}) = \mathbf{u}_0, \end{aligned}$$

for the bounded measurable functions $u_{j0}$, $j=1,\dots ,n$.

The standard theory of hyperbolic conservation laws is concerned with solutions which are at worst locally integrable. More precisely, if the system is *strictly hyperbolic* and *genuinely nonlinear*^1^, and if the total variation of $\mathbf{u}_0$ is small enough, then the Cauchy problem has a solution [5, 6, 21, 39, 41, 42]. If the initial functions are step functions, then the Cauchy problem is called the Riemann problem. If the differences in function values at the jumps are small then the Riemann problem can be solved uniquely using Lax-admissible solutions consisting of rarefaction waves, compressive shock waves and contact discontinuities.

On the other hand, if the above conditions are not fulfilled, then the Cauchy, or even the Riemann problem may not admit a Lax admissible weak solution or even any weak solution. Indeed, the standard theory is far from complete, as even some fairly simple problems cannot be resolved in a satisfactory manner (see e.g. [13, 16, 35, 37, 45]). Nevertheless, one might expect that in the case of physically relevant systems (such as e.g. the *p*-system arising in gas dynamics and nonlinear elasticity) one should be able to prove existence of weak solutions of bounded variation (BV) of the corresponding Cauchy problem. On the other hand, in fairly recent contributions [8, 64] where the *p*-system was considered, it was shown that the Glimm scheme and wave front tracking, respectively, for a Cauchy problem with large initial data blow up (in the sense that uniform BV bounds on the approximate solutions are not available). However, note that for certain functions *p*, Cauchy problems for the *p* system can be resolved [4].

In order to deal with situations where the standard theory fails, expanding the space of possible solutions to the space of Radon measures has been useful in some cases. In particular, if solutions are allowed to contain Dirac $\delta$-distributions, some systems to which the standard theory does not apply can be resolved. The first result in this direction can be found in [37]. However, the interest in such an approach (as well as its necessity) was particularly raised after it was shown in [35] that strictly hyperbolic and genuinely nonlinear system of conservation laws with compact Hugoniot locus feature these non-standard solutions.

The question which naturally appears is how to incorporate the $\delta$-distribution as a part of solution to (1.1). If we substitute the distribution directly into (1.1), the problem of multiplication of singular distributions arises. It turns out that when one deals with systems which are linear with respect to one of the unknowns (and since we can approximate any locally integrable initial data by a step function), it is sufficient to understand the multiplication of a $\delta$ distribution and a Heaviside function (see [61] and references therein).

Let us focus therefore on the issue of defining the multiplication of a $\delta$-distribution and a Heaviside function. There are several reasonable ways to define such a product, but we shall mention only two main approaches. The first one is to define $\int H(x)\delta (x) \, dx = H(0)$ which means that one needs to define the value of the Heaviside function at the jump point. Such an approach has been used in [13, 28] to introduce the notion of measure-type solution for (1.1). The framework introduced in [28] yields uniqueness of solutions if an additional condition of Oleinik-type is required. However, that approach can be applied only to equations which are linear with respect to one of the unknowns, as already alluded to above.

The other main approach to making sense of singular solutions is the weak asymptotic method which entails defining smooth approximations $(\delta _\varepsilon )$ and $(H_\varepsilon )$, and considering the product $(\delta _\varepsilon H_\varepsilon )$. The limit as $\varepsilon \rightarrow 0$ of the family $(\delta _\varepsilon H_\varepsilon )$ can then be defined to be the product of the $\delta$-distribution in the context of the weak asymptotic method, and one obtains approximate solutions to (1.1) for which one concludes that they converge weakly to a measure containing a $\delta$-distribution. This approach has been used in [14, 16, 65] among many others, and is similar to the vanishing viscosity and the vanishing pressure methods used for example in [29, 35, 45]. One may also note that the family $(\delta _\varepsilon H_\varepsilon )$ can be considered as an element of a Colombeau algebra, such as defined in [11]. This approach has proved fruitful in some cases (see [46] for example). In this direction, we also mention the $\alpha$-product by Sarricco (see e.g. [51, 52, 57, 58] and references therein) which is applied in the case of various systems of conservation laws and similar equations and which works in the case of fully nonlinear equation (i.e. nonlinear with respect to all unknowns).

Close examination of the weak asymptotic method reveals that is does not give precise meaning to whether and how the limiting distributions satisfy the original differential equations. This was first achieved in the work by Danilov and Shelkovich [17] where a variational formulation for the $\delta$-type framework was proposed for systems which are linear with respect to one of the unknown functions and for $\delta$-solutions where components containing the $\delta$-distributions are supported on isolated curves. Indeed, the same idea can be used to define $\delta$-solutions for general $2\times 2$ systems of conservation laws, such as explained in [30]. In the present contribution, we extend the variational formulation to the case of $n\times n$ systems such as (1.1) and we prove that the Riemann problem for this system always has a solution in the framework of the definition given. We then extend the existence theorem to the general Cauchy problem by using a front-tracking approach. The key step is to estimate the BV norm of the solution after each interaction, and this is facilitated by introducing the singular parts of the solution.

The variational formulation of the Cauchy problem is very weak, and uniqueness of solutions cannot be expected. In fact, it can be shown that even the Riemann problem for a $2\times 2$ system cannot be solved uniquely in the context of the variational formulation of singular solutions (see [30]). We partially deal with this issue by formulating an admissibility criterion which while not providing uniqueness at least eliminates a wide range of possible trivial solutions. The admissibility criterion is physically motivated and requires that at least one of the equations of the system satisfies the Rankine–Hugoniot condition at every shock.

Finally, it is shown that at least in the special case of the Riemann problem and a polynomial flux function, the solution can be obtained from the weak asymptotic method satisfying the admissibility conditions. This result establishes that the solution stems from an approximation procedure within the framework of smooth approximate solution converging towards $\delta$-type solutions which is similarly in nature the above mentioned vanishing viscosity or vanishing pressure approaches. This tool enables us to make the connection between Rankine–Hugoniot deficits as appearing in the variational formulation, and the $\delta$-type solutions to the original system.

As the results proved here do not rely on the assumption of hyperbolicity, the impression may arise that the solution concept provided here is too broad to be useful. We partially agree with such an assessment, but we also note that so far there are no general results for solvability of systems of the form (1.1), (1.2) in the literaure (except for some very special situations). In addition, our contribution is not an attempt to solve the problem in the standard space of locally integrable functions, but to investigate an alternative approach which would eventually lead to a satisfactory solution to (1.1), (1.2) from both the physical and the mathematical point of view. Finally, as shown in Sect. 3, we are able to find approximate smooth solutions to Riemann problems for a wide range of conservation laws which converge towards the $\delta$-type solutions introduced here.

The paper is organized as follows. The definition of the variational formulation, admissibility conditions, and the existence theorem are given in Sect. 2. Sect. 3 contains a detailed account of the application of the weak asymptotic method to the Riemann problem for a $2\times 2$ system. Finally, in Sect. 4, we review the physical validity of singular solutions of conservation laws and show how the present approach arises naturally in the context of the the shallow-water system.

## Variational Formulation of Delta-Shock Solutions

In this section, we shall introduce a solution framework in which any Cauchy problem corresponding to (1.1) with *BV*-initial data has a solution. It is based on the variational formulation of $\delta$-shock solutions introduced in [17] in the case of $2\times 2$ systems which are linear with respect to one of the unknowns. Note that in such systems the $\delta$ distribution arises as a part of the solution defined along characteristics [16] as well as in the limit of the vanishing viscosity approximations [17, 35]. As shown in [31], the variational formulation may also be applied to to arbitrary $2\times 2$ systems of conservation laws, and this extension may be justified using the complex weak asymptotic method. However, in general this variational notion of solution is so weak that uniqueness can only be proved in special cases. In the following, we will generalize the variational formulation to $n\times n$ systems of conservation laws and Cauchy data, such as given by (1.1), (1.2).

As a first step, we shall define $\delta$-type solutions for Riemann problems corresponding to systems of conservation laws. Suppose $\Gamma$ is a graph in the closed upper half plane containing a single Lipschitz continuous arc $\gamma$. Let $\Gamma _0 = \{x_0\}$ be the initial point of the arc $\gamma$. Let $u_k$, $k=1,\dots ,n$, be distributions of the form

$$\begin{aligned} u_k(t,x)&=U_k(t,x) + \alpha _k(t,x)\delta (\gamma ), \end{aligned}$$

where $U_k \in L^\infty ({\mathbb {R}}_+ \times {\mathbb {R}})$ and $\alpha _k(t,x)$ are real-valued functions. Let $\frac{\partial \varphi (t,x)}{\partial \mathrm{l}}$ denote the tangential derivative of a function $\varphi$ on the graph $\gamma$, and let $\int _{\gamma }$ denote the line integral over the arc $\gamma$.

### Definition 2.1

A graph $\Gamma$ and a sequence of distributions $(u_1,\dots ,u_d)$ of the form

2.1 $$\begin{aligned} u_k(t,x)=U_k(t,x)+ \alpha _k(t,x) \delta (\gamma ), \end{aligned}$$

with $U_k\in L^\infty ({\mathbb {R}}_+ \times {\mathbb {R}})$, $\alpha _k \in C^1(\Gamma )$, $k=1,\dots ,n$, is called a generalized $\delta$-shock wave solution of system (1.1) with initial data $u_0(x)+ \alpha _k(0,x_0)\delta \left( x-x_0 \right)$, $k=1,\dots ,n$, if the integral identities

2.2 $$\begin{aligned}&\int _{{\mathbb {R}}_+}\! \! \int _{{\mathbb {R}}}\left( U_k \partial _t\varphi +f_k(U_1,\dots ,U_d) \partial _x\varphi \right) dxdt+\int _{{\mathbb {R}}}U_{0k}(x)\varphi (0,x)~dx, \qquad \nonumber \\&\qquad + \int _{\gamma } \alpha _k(t,x){ \textstyle \frac{\partial \varphi (t,x)}{\partial \mathbf{l}}} + \alpha _k(0,x^0) \varphi (0,x^0)=0, \end{aligned}$$

hold for all test functions $\varphi \in \mathcal{D}({\mathbb {R}}\times {\mathbb {R}}_+)$ and for $k=1,\dots ,n$.

First note that if we assume $\gamma =\{(t,\gamma (t)): \; t\in [0,T] \}$, we can also assume that $\varphi (T,x) \ne 0$ in which case one should add the terms $- \int _{\gamma }\alpha _k(T,\gamma (T))\varphi (T,\gamma (T))$ on the left-hand sides of (2.2). Also notice that (2.2) represents a generalization of the classical weak solution concept for (1.1). Indeed, if we omit the integral over the curve $\gamma$, we reach the standard variational formulation of (1.1). However, Definition 2.1 is very weak in the sense that a wide range of distributions satisfy its requirements. This is not surprising since even in the case of the standard weak solutions for scalar conservation laws, non-uniqueness issues arise [6].

Let us first look at the system (1.1) with singular Riemann data

2.3 $$\begin{aligned} u_k(0,x)=\alpha ^k_0 \delta (x) + {\left\{ \begin{array}{ll} u^k_L, &{} x<0, \\ u^k_R, &{}x>0. \end{array}\right. } \end{aligned}$$

Using Definition 2.1, it is not difficult to see that for any $c\in {\mathbb {R}}$, and any given initial states $\mathbf{u}_L=(u^1_L,\dots ,u_L^d)$ and $\mathbf{u}_R=(u^1_R,\dots ,u_R^d)$, a solution of the form

$$\begin{aligned} \mathbf{u}(t,x)=\mathbf{U}(t,x)+{\varvec{\alpha }}(t) \delta (x-ct), \end{aligned}$$

exists, where $\mathbf{U}(t,x) =(U_1(t,x),\dots ,U_d(t,x))$ is given by

2.4 $$\begin{aligned} \mathbf{U}(t,x)={\left\{ \begin{array}{ll} \mathbf{u}_L, &{} x<ct, \\ \mathbf{u}_R, &{}x>ct, \end{array}\right. } \end{aligned}$$

and the amplitude ${\varvec{\alpha }}(t)=(\alpha _1(t),\dots ,\alpha _d(t))$ of the singular part of the shock is given by

2.5 $$\begin{aligned} {\varvec{\alpha }}(t) =\left( c[\mathbf{U}]-[{{\mathfrak {f}}}] \right) t +{\varvec{\alpha }}(0) := \left( c[\mathbf{u}_R - \mathbf{u}_L]-[{{\mathfrak {f}}}(\mathbf{u}_R) - {{\mathfrak {f}}}(\mathbf{u}_L)] \right) t+{\varvec{\alpha }}(0), \end{aligned}$$

where ${{\mathfrak {f}}}=(f_1,\dots ,f_d)$. In other words, the following theorem holds.

### Theorem 2.1

Given any constant vectors $\mathbf{u}_L, \mathbf{u}_R \in {\mathbb {R}}^d$, and given any $c\in {\mathbb {R}}$, define the distributions $\mathbf{u}(t,x)=\mathbf{U}(t,x)+{\varvec{\alpha }}(t) \delta (x-ct)$ where $\mathbf{U}(t,x)$ is given by (2.4), and ${\varvec{\alpha }}(t)$ is given by (2.5). Then $\mathbf{u}(t,x)$ is a solution of the Riemann problem (1.1), (2.3) in the sense of Definition 2.1.

### Proof

The proof of the theorem follows by substituting $\mathbf{u}$ into (2.2) (for simplicity, we shall assume here and in the sequel $T=\infty$ unless stated otherwise). After standard transformations, we reach the identities

2.6 $$\begin{aligned}&\int _{{\mathbb {R}}_+}\left( c[\mathbf{U}]-[{{\mathfrak {f}}}(\mathbf{U})]\right) \varphi (t,ct)~dt -\int _{{\mathbb {R}}_+}{\varvec{\alpha }}'(t)\varphi (t,ct)~dt= 0. \end{aligned}$$

From here, the statement of the theorem follows immediately. $\square$

Before introducing admissibility conditions which will eliminate trivial situations given by the previous theorem, we will define a solution framework which can be applied to any Cauchy problem associated to (1.1). Notice that if we assume that the curves from the Definition 2.1 are straight lines of the form $\gamma _i=\{x=c_i (t-t_i)+x_i\}$, then the relation (2.2) simplifies in the sense that instead of the tangential derivative appearing there, we simply have the expression (assume that $\mathrm{supp \varphi }\subset (0,\infty )$)

2.7 $$\begin{aligned} \int _{\gamma _i}\frac{\partial \varphi }{\partial l} \, {\varvec{\alpha }}(t)= \int _{t_i}^\infty {\varvec{\alpha }}(t) \frac{\partial \varphi (t,x_i+c_i (t-t_i))}{\partial t} =-\int _{t_i}^\infty {\varvec{\alpha }}'(t) \varphi (t,x_i+ c (t-t_i))dt.\nonumber \\ \end{aligned}$$

Moreover, if we assume that we are solving the Riemann problem, then we can also assume that ${\varvec{\alpha }}'$ are constants (at least in the intervals between possible waves interactions). With this example in mind, we introduce a subspace of the dual to the Bochner space $L^1([0,T];C_0({\mathbb {R}}))$ for a given $T>0$ as follows.

### Definition 2.2

Let $T>0$ be given, and let $C_0({\mathbb {R}})$ be the space of continuous functions on ${\mathbb {R}}$ which decay to 0 at infinity. We denote by $\mathcal{G}_e(T)$ the subspace of the dual of the Bochner space $L^1([0,T];C_0({\mathbb {R}}))$ given by

$$\begin{aligned} \mathcal{G}_e(T)&=\left\{ \sum \limits _{k=1}^{N} \alpha _k(t)\delta (x-x_{k}) \text{ for } \alpha _k \in C_c([0,T)), \, x_{k}\in {\mathbb {R}}\right\} . \end{aligned}$$

It can be proved that the closure of $\mathcal{G}_e(T)$ with respect to the weak$^\star$ topology actually coincides with the entire dual space $L^1([0,T];C_0({\mathbb {R}}))^\star = L^\infty _{w^*}([0,T];\mathcal{M}( {\mathbb {R}}))$, where $\mathcal {M}({\mathbb {R}})$ is the space of real-valued finite Radon measures. Indeed, we have the following proposition.

### Proposition 2.1

We have $Cl\left( \mathcal{G}_e(T)\right) =L^1([0,T];C_0({\mathbb {R}}))^\star$, where the closure *Cl* is understood with respect to the weak$^\star$ topology.

### Proof

Let $\mu \in L^1([0,T];C_0({\mathbb {R}}))^\star$ be arbitrary. We construct an approximation as follows. For a given $n {\mathbb {N}}$, we introduce a uniform partition of the interval $[-n,n]$ by setting $x^n_k = \frac{k}{n}$, $k = -n^2, \cdots , n^2$. We then define

$$\begin{aligned} \mu _n(t,x) = \sum \limits _{k = -n^2}^{n^2} \mu (t,[x^n_k,x^n_{k+1}))\delta (x-x^n_k). \end{aligned}$$

Since $\mu (t,\cdot )$ has finite variation, and indeed, $\sup _{t\ge 0}|\mu (t,\cdot )| < \infty$, the sequence $\mu _n(t,x)$ is bounded in $L^\infty _{w^*}([0,T];\mathcal{M}( {\mathbb {R}}))$. Thus, to prove weak convergence, it is sufficient to consider test functions from a dense subspace. In particular, we can show that

2.8 $$\begin{aligned} _{ L^{\infty }([0,T];\mathcal {M}({\mathbb {R}})) }\langle (\mu -\mu _n),\varphi \rangle _{L^1([0,T];C_0({\mathbb {R}}))} \rightarrow 0 \quad \text{ as } \quad n \rightarrow \infty \end{aligned}$$

for any $\varphi \in L^1([0,T];C_c({\mathbb {R}}))$, i.e. $\varphi$ with compact support in *x*. In fact we have

$$\begin{aligned}&_{L^\infty ([0,T];\mathcal{M}( {\mathbb {R}}))}\langle (\mu -\mu _{n}),\varphi \rangle _{L^1([0,T];C_0({\mathbb {R}}))}\\&= \int _0^T \sum \limits _{k=-n^2}^{n^2} \int _{x_k^n}^{x_{k+1}^n} \varphi (t,x) d \mu (t,x) dt \\ {}&\ \ - \int _0^T \sum \limits _{k=-n^2}^{n^2} \varphi (t,x_k^n) \mu \left( t,[x_k^n, x_{k+1}^n)\right) dt + \int _{{\mathbb {R}}^+\times [-n,n]^C}\varphi (t,x)d\mu (t,x), \end{aligned}$$

and since $\varphi$ has compact support, the last term in the expression above is zero for large enough *n*. Moreover, such $\varphi (t,x)$ is uniformly continuous in (*t*, *x*), and can be approximated by a function taking a finite number of values (a simple function). Given a challenge number $\varepsilon$, we can take *n* large enough such that $|\varphi (t,x) - \varphi (t,x_k^n)| < \varepsilon$ so long as $x \in [x_k^n, x_{k+1}^n)$. Since the term $\varphi (t,x_k^n) \mu \left( t,[x_k^n, x_{k+1}^n)\right)$ is simply the definition of the integral $\int _{x_j^n}^{x_{j+1}^n} \varphi (t,x_j^n) \chi _{[x_j^n, x_{j+1}^n)}(x) d\mu (t,x)$ of the function $\varphi (t,x_j^n) \chi _{[x_j^n, x_{j+1}^n)}(\cdot )$, we then have

$$\begin{aligned}&\Big | \sum \limits _{j=-n^2}^{n^2} \int _{x_j^n}^{x_{j+1}^n} \varphi (t,x) d \mu (t,x) - \sum \limits _{j=-n^2}^{n^2} \varphi (t,x_j^n) \mu \left( t,[x_j^n, x_{j+1}^n)\right) \Big | \\&\quad \le \sum \limits _{j=-n^2}^{n^2} \int _{x_j^n}^{x_{j+1}^n} \big | \varphi (t,x) - \varphi (t,x_j^n) | d \mu (t,x) \\&\quad \le \sum \limits _{j=-n^2}^{n^2} \int _{x_j^n}^{x_{j+1}^n} \varepsilon \, d \mu (t,x) \\&\quad = \varepsilon \sum \limits _{j=-n^2}^{n^2} \mu \left( t,[x_j^n, x_{j+1}^n)\right) . \end{aligned}$$

Since $\mu$ has bounded variation, we have

$$\begin{aligned} _{L^\infty ([0,T];\mathcal{M}( {\mathbb {R}}))}\langle (\mu -\mu _{n}),\varphi \rangle _{L^1([0,T];C_0({\mathbb {R}}))} \le T \varepsilon \sup _{0 \le t \le T}|\mu (t,\cdot )| \end{aligned}$$

for large enough *n*, and this concludes the proof. $\square$

We next want to define an operation similar to (2.7) for elements of $L^\infty _{w^*}([0,T];\mathcal{M}({\mathbb {R}}))$. To this end, we assume that $\mu \in L^\infty _{w^*}([0,T];\mathcal{M}({\mathbb {R}}))$ is weakly differentiable, i.e. that $\mu (\cdot , {\mathcal {A}})$ is differentiable for a given measurable set ${\mathcal {A}}$. We use a partition $\{x^n_k \}$ as in the previous proposition. We then take the following approximation of the measure $\mu$ by elements of ${\mathcal {G}}_e$:

2.9 $$\begin{aligned} \mu _n(t,x)=\sum \limits _{k=-n^2}^{n^2} \mu (t,[x_k^n,x_{k+1}^n)) \delta (x-k_k^n) =\sum \limits _{k=-n^2}^{n^2} \alpha ^n_k(t) \delta (x-x_k^n). \end{aligned}$$

We can now introduce the following definition.

### Definition 2.3

We say that the functional $\mu _l=\partial _l \mu$ is a generalized tangential derivative of $\mu \in L^\infty _{w^*}([0,T];\mathcal{M}({\mathbb {R}}))$ if along some subsequence for every $\varphi \in C_c^1([0,T]\times {\mathbb {R}})$:

2.10 $$\begin{aligned} \lim \limits _{n \rightarrow \infty } \int _{{\mathbb {R}}_+}\sum \limits _{k=-n^2}^{n^2} \partial _t \alpha ^n_{k}(t) \varphi (t,x^n_k) dt=\int _{{\mathbb {R}}_+}\langle \mu _l,\varphi (t,\cdot ) \rangle dt \end{aligned}$$

where $\alpha ^n_k$ are given by (2.9).

### Remark 1

We note that in the case when the measure has the form

$$\begin{aligned} \mu (t,x)=\sum \limits _{j\in {\mathbb {Z}}} \alpha _j(t)\delta (x-x_j-\beta _j(t)) \end{aligned}$$

then the corresponding tangential derivative has the form

$$\begin{aligned} \mu _l(t,x)=\sum \limits _{j\in {\mathbb {Z}}} \alpha '_j(t)\delta (x-x_j-\beta _j(t)) \end{aligned}$$

i.e. for every $$\varphi \in C_c^1([0,T]\times {\mathbb {R}})$$

2.11 $$\begin{aligned} \int _{{\mathbb {R}}_+}\langle \mu _l,\varphi (t,\cdot ) \rangle dt = \int _{{\mathbb {R}}_+} \sum \limits _{j\in {\mathbb {Z}}} \alpha '_j(t)\varphi (t,x_j+\beta _j(t))dt. \end{aligned}$$

Indeed, by the definition of the generalized tangential derivative, we need to approximate the measure $\mu$ by the atomic measures of the form $\mu _n(t,x)=\sum \nolimits _{k=-n^2}^{n^2} \alpha ^n_k(t)\delta (x-x^n_k)$ where $\alpha _k^n(t)=\mu (t,[x_k^n,x_{k+1}^n))$. Clearly, we have

$$\begin{aligned} \alpha _k^n = \alpha _j, \ \text{ if } \ x_j +\beta _j(t) \in [x_k^n,x_{k+1}^n) \end{aligned}$$

from where we see that

$$\begin{aligned} \lim \limits _{n \rightarrow \infty } \int _{{\mathbb {R}}_+}\sum \limits _{k=-n^2}^{n^2} \partial _t \alpha ^n_{k}(t) \varphi (t,x^n_k) dt= \int _{{\mathbb {R}}_+}\sum \limits _{j\in {\mathbb {Z}}} \alpha '_j(t) \varphi (t,x_j+\beta _j(t))dt, \end{aligned}$$

and (2.11) follows directly.

Clearly, $m_l$ will always exist since a bounded sequence in $L^\infty _{w^*}([0,T];\mathcal{M}({\mathbb {R}}))$ is weakly precompact. On the other hand, such $m_l$ might not be unique since different subsequences could converge to different limits. So while the tangential derivative in general is not well defined, fixing the approximation in the from (2.9) reduces the number of possible generalized tangential derivatives significantly.

A simple example is the $\delta$-distribution of the form $\alpha (t) \delta (x-ct)$. In this case, it is straightforward to see that the generalized tangential derivative should be $\alpha '(t)\delta (x-ct)$ and that it is the unique tangential derivative of $\alpha (t) \delta (x-ct)$. As a more interesting example, let us compute the tangential derivative for a measure defined by a regular function. Since the $\delta$-distribution is the distributional derivative of a shock wave, an interesting case will be the measure defined by the derivative of a rarefaction wave. We stress that we are not solving the conservation law given in the example below, but merely compute the tangential derivative of a measure. A solution to the scalar conservation law does not contain any special structures as part of the solution (see Definition 2.4 below), they are, as well known, ordinary locally integrable functions.

### Example 1

Rarefaction waves are given by the functions of the form $u(\frac{x}{t})$ which, in the scalar case, solve the equation

$$\begin{aligned} \partial _t u +\partial _x f(u)=0. \end{aligned}$$

Denote by *m* the measure defined by the function $\partial _x u(\frac{x}{t})=\frac{1}{t} u'(\frac{x}{t})$:

$$\begin{aligned} m(t,x)= u\left( { \textstyle \frac{x}{t}}\right) . \end{aligned}$$

Let us compute $\partial _l m$. According to the definition of the generalized tangential derivative, we consider the approximation

$$\begin{aligned} m_n(t,x)=\sum \limits _{k=-n^2}^{n^2} m(t,[x_k^n,x_{k+1}^n)) \delta (x-x^n_k). \end{aligned}$$

The tangential derivative is a weak limit (along a subsequence) of $\partial _t m_n(t,x)$. We have

$$\begin{aligned} \partial _t m_n(t,x)&=\sum \limits _{k=-n^2}^{n^2} \partial _t m(t,[x_k^n,x_{k+1}^n))\delta (x-x^n_k) = \sum \limits _{k=-n^2}^{n^2} \int _{x_k^n}^{x_{k+1}^n} \partial _t u\left( { \textstyle \frac{x}{t}}\right) dx \, \delta (x-x^n_k) \\&=-\sum \limits _{k=-n^2}^{n^2} \int _{x_k^n}^{x_{k+1}^n}\partial _x f(u({ \textstyle \frac{x}{t}}))dx \, \delta (x-x_k^n) \end{aligned}$$

which converges weakly toward $\partial _x f(u(\frac{x}{t}))$. From the above, we conclude that the generalized tangential derivative of *m* is

$$\begin{aligned} \partial _l m=-\partial _x f \left( u \left( { \textstyle \frac{x}{t}} \right) \right) . \end{aligned}$$

With these preliminaries in place, we can introduce $L^\infty _{w^*}([0,T];\mathcal{M}({\mathbb {R}}))$-solutions to an arbitrary Cauchy problem corresponding to (1.1).

### Definition 2.4

We say that the pair of distributions

2.12 $$\begin{aligned} \mathbf{u}(t,x)&=(\mathbf{U}(t,x), \mathbf{m}(t,x)):=\mathbf{U}(t,x)+ \mathbf{m}(t,x), \end{aligned}$$

where $\mathbf{U}(t,x) =(U_1(t,x),\dots ,U_d(t,x))$ with $U_i \in L^{\infty }({\mathbb {R}})$ and $\mathbf{m}=(m_1,\dots ,m_d)$ weakly differentiable in $L^\infty _{w^*}([0,T];\mathcal{M}({\mathbb {R}}))^d$, is a measure-type solution to (1.1) with the initial data $\mathbf{u}|_{t=0}=\mathbf{u}_0$ if the following relations hold for any $\varphi \in C^1_c({\mathbb {R}}_+\times {\mathbb {R}})$

2.13 $$\begin{aligned}&\int _{{\mathbb {R}}_+}\! \! \int _{{\mathbb {R}}}\left( \mathbf{U}\partial _t\varphi +{{\mathfrak {f}}}(\mathbf{U}) \partial _x\varphi \right) dxdt + \int _{{\mathbb {R}}}\mathbf{u}_0(x)\varphi (x,0)~dx \qquad \nonumber \\&\qquad - \int _{{\mathbb {R}}_+} \langle \partial _l \mathbf{m}, \, \varphi (\cdot ,t) \rangle dt =0 \end{aligned}$$

for some generalized tangential derivatives $\partial _l \mathbf{m}=(\partial _l m_1,\dots ,\partial _l m_d)$ of the measure $\mathbf{m}$.

As is obvious from the formulation of the functionals $\mathbf{m}$ and $\frac{\partial \mathbf{m}}{\partial l}$, this definition is a generalized version of Definition 2.1. Indeed, Definition 2.4 reduces to Definition 2.1 if the $\delta$-distributions involved in the solution to (1.1) are supported on isolated smooth curves (see (2.7)).

However, Definition 2.4 is quite general as for any initial data $u_0\in BV({\mathbb {R}})$ and any fixed speed vector *c*, the distribution

2.14 $$\begin{aligned} \mathbf{U}(t,x)+ \mathbf{m}(t,x)=u_0(x-ct)+t(cu_0'-f(u_0)')\delta (x-ct) \end{aligned}$$

represents a measure-type solution to (1.1). To reduce the number of possibilities, we introduce basic admissibility conditions which will not provide uniqueness of this type of solution, but which eliminates pathological solutions such as (2.14).

### Definition 2.5

We say that the measure-type solution to (1.1) satisfies basic admissibility conditions if it represents a weak limit to the following sequence of measures

2.15 $$\begin{aligned} \mathbf{u}_n(t,x)=\mathbf{U}^n(t,x) + \sum \limits _{j\in {\mathbb {Z}}} {\varvec{\alpha }}^n_j(t) \delta (x-x^n_j - c^n_{j}(t-t^n_{j})) \chi _{[t_j^n,T_j^n]}(t), \end{aligned}$$

where $0\le t_j^n \le T_j^n$ for every $n,j \in {\mathbb {N}}$, ${\varvec{\alpha }}^n_j(t)=(\alpha ^n_{1j},\dots ,\alpha ^n_{dj})(t)$ are affine functions, and $\mathbf{U}^n(t,x)=(U_1^n(t,x),\dots ,U_d^n(t,x))$ are step functions such that at each step at least one of the equations in system (1.1) is satisfied by $\mathbf{U}^n(t,x)$ in the standard weak sense (i.e. the corresponding Rankine–Hugoniot conditions are satisfied).

The latter definition is motivated by the wave front tracking procedure. Actually, using such an approach, we are able to prove that there exists the measure-type solution to any Cauchy problem corresponding to (1.1) with BV-initial data satisfying the basic admissibility conditions^2^. For simplicity, we shall provide the proof in the case of $2\times 2$ system. The proof is analogous in the case of an $n\times n$ system, and it is based on BV-estimates which do not increase during the interactions. Instead, we have to increment the strength of $\delta$-functions appearing during the interaction, but the increment is also finite. We note also that we use Remark 1 in the course of the proof of the next theorem.

### Theorem 2.2

Let $n=2$, and assume that $f_1,f_2 \in C^1({\mathbb {R}}^2)$ and $u_{10},u_{20} \in L^\infty ({\mathbb {R}}) \cap BV({\mathbb {R}})$. Then, the system (1.1) with the initial data $u_1|_{t=0}=U_{10}$, $u_2|_{t=0}=U_{20}$ admits a solution in the sense of Definition 2.4 satisfying the basic admissibility conditions.

### Proof

We shall apply the method of wave front tracking [6, 27]. In the framework of that method, one approximates the initial data by piecewise constant functions and then solves Riemann problems at each step. We thus obtain new waves which mutually interact. In the case of the standard wave front tracking approach which implies solving a Riemann problem using Lax admissible shock waves, the BV-character of the solution can be lost as the result of the interactions of different waves (the solution can blow up [7, 64]).

Using our definition, it is not difficult to construct approximate solution for an arbitrary Cauchy problem using the wave front tracking algorithm. Instead of a possible increase of the local BV-bounds, we will obtain $\delta$-type solutions, and the coefficient accompanying the $\delta$-function will fix the Rankine–Hugoniot deficit.

As we have seen in the proof of Theorem 2.1, when solving a Riemann problem, we can choose practically any speed of the shock which solves the problem. We correct the mistake originating from the Rankine–Hugoniot conditions using the $\delta$-shocks. What is reasonable to do is to minimize the strength of the $\delta$-shocks (since in this way we get a smaller error with respect to the standard situation when we use the weak solution concept). The correction will be smaller if the speed of the shock is smaller. In other words, we shall use the following procedure:

(A) We approximate the functions $U_{01}$ and $U_{02}$ by the piecewise constant functions
  $$\begin{aligned}&U_{01}^N(x)=\sum \limits _{j\in {\mathbb {Z}}} u_1^j \chi _{ \{ (x_j,x_{j+1}) \} }(x), \\&U_{02}^N(x)=\sum \limits _{j\in {\mathbb {Z}}} u_{2}^j \chi _{ \{(x_j,x_{j+1})\} }(x), \end{aligned}$$
  where $\chi _{ \{(x_j,x_{j+1}) \} }$ is the characteristic function on the interval $(x_j, x_{j+1})$ defined in the proof of Proposition 2.1, and we require $\Vert U_{01} - U_{01}^N\Vert _{L^1(K)} = o(1)$, $K\subset \subset {\mathbb {R}}$, for $i=1,2$. For $j \in {\mathbb {Z}}$, denote by
  $$\begin{aligned} C_i^j=\frac{f_i(u_{1}^{j+1},u_{2}^{j+1})-f_i(u_{1}^{j},u_{2}^j)}{u_{i}^{j+1}-u_{i}^j}, \ \ i=1,2 \end{aligned}$$
  the speeds given by the Rankine–Hugoniot conditions of the first and second equations ($i=1,2$).
(B) We thus obtain a series of Riemann problems; one for the boundary of each interval from (A) which we solve using Definition 2.1 as follows. Denote by $\alpha _i^j$, $i=1,2$, $j = 1 \dots N$, the corresponding Rankine–Hugoniot deficits (see (2.5)) given as follows:
  $$\begin{aligned} ( \alpha _1^j)':=\partial _t \alpha _1^j = c_j(u_{1}^{j+1}-u_{1}^j) - \left[ f_1(u_{1}^{j+1},u_{2}^{j+1})-f_1(u_{1}^{j},u_{2}^{j}) \right] , \end{aligned}$$
  $$\begin{aligned} ( \alpha _2^j)':=\partial _t \alpha _2^j = c_j(u_{2}^{j+1}-u_{2}^j) - \left[ f_2(u_{1}^{j+1},u_{2}^{j+1})-f_2(u_{1}^{j},u_{2}^{j}) \right] . \end{aligned}$$
  The corresponding speed $c_j$, appearing in the place of *c* from (2.6), satisfies
  $$\begin{aligned} c_j= {\left\{ \begin{array}{ll} C^j_1, &{} |(\alpha _1^j)'| \le |(\alpha _2^j)'| \\ C^j_2, &{} else \end{array}\right. } \end{aligned}$$
  and the correction ($\delta$-distribution) will be adjoined to the function $U_{0i}$ for which $|(\alpha _i^j)'|=\min \{|(\alpha _1^j)'|,|(\alpha _2^j)'| \}$. In this way, we ensure that the strength of the $\delta$-distribution appearing as a part of the approximate solution will be as small as possible and we still have the Rankine–Hugoniot conditions satisfied for at least one of the equations of the system.
(C) We obtain a family $(u_1^N,u_2^N)$ of elements from $Lip([0,T];\mathcal{M}({\mathbb {R}}))_e$ given by (2.16) whose weak limit is in $Lip([0,T];\mathcal{M}_{loc}({\mathbb {R}}))$, and which represents the solution to the considered Cauchy problem in the sense of Definition 2.4.

Items A) and B) are standard in wave front tracking. As for the item C), we use Theorem 2.1. More precisely, we solve each of the Riemann problems so that we adjoin a $\delta$-distribution either to $u_1$ or to $u_2$ (or to both $u_1$ and $u_2$) so that the speed of the corresponding $\delta$-shock is minimal and that, at the same time, it is given by the Rankine–Hugoniot conditions of one of the equations.

**Fig. 1:**
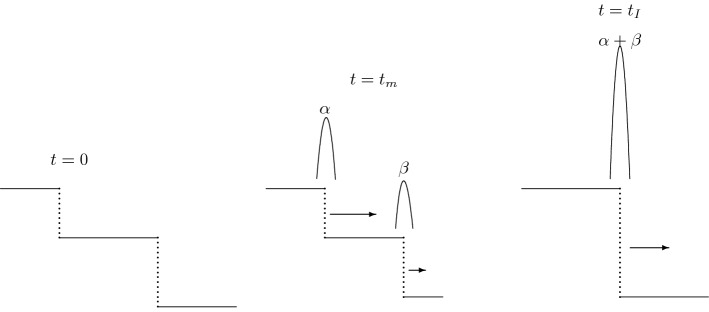
Evolution of two shock waves. At $t_m$ we have two $\delta$-distributions with the strengths $\alpha$ and $\beta$. They all merge at the moment $t_I$

We thus obtain the solution $(u_1^N, u_2^N)$ of the approximate problem until the set of first interactions. The interaction means that the shock waves (together with the accompanying $\delta$-distributions) are at the same point in ${\mathbb {R}}_+\times {\mathbb {R}}$. At that moment, the shocks and $\delta$-distributions will merge (the state between two shocks will disappear and $\delta$ will have the strength equal to the strength of the interacting $\delta$-s at the moment of interaction; see Fig. 1).

At that moment, we stop the time and solve a new set of Riemann problems as done in Theorem 2.1 (this time involving the $\delta$-distributions as part of initial data). Thus, for any $t \in [0,T]$, we conclude that the distributions $u^\varepsilon _1$ and $u^\varepsilon _2$ have the form

2.16 $$\begin{aligned} \begin{aligned} u_1^N(t,x)=U^N_1(t,x) + \sum \limits _{j} \alpha _1^j(t) \delta (x-x_j - c_{j}(t-t_{j})) \chi _{(t_j,T_j)}(t)\\ u_2^N(t,x)=U^N_2(t,x)+\sum \limits _{j} \alpha _2^j(t) \delta (x-x_j-c_j(t-t_j)) \chi _{(t_j,T_j)}(t), \end{aligned} \end{aligned}$$

for some piecewise constant functions $U^N_1$ and $U^N_2$, and where $t_j$ denotes the time of creation of the singularity, and $T_j$ denotes the time of the next interaction. The solution satisfies the relations

2.17 $$\begin{aligned}&\int _{{\mathbb {R}}_+}\! \! \int _{{\mathbb {R}}}\left( U_1^N \partial _t\varphi +f_1(\mathbf{U}^N) \partial _x\varphi \right) dxdt +\int _{{\mathbb {R}}}U_{01}^N(x)\varphi (x,0)~dx, \nonumber \\&\quad - \sum \limits _{j} \int _{{\mathbb {R}}_+} \partial _t \alpha _1^{j}(t) \varphi \left( x_{j}+c_{j}(t-t_{j}),t \right) dt=0, \qquad \qquad \qquad \end{aligned}$$

and

2.18 $$\begin{aligned}&\int _{{\mathbb {R}}_+}\! \! \int _{{\mathbb {R}}}\left( U_2^N \partial _t \varphi + f_2(\mathbf{U}^N)\partial _x\varphi \right) ~dxdt + \int _{{\mathbb {R}}}U_{02}^N(x)\varphi (x,0)~dx \nonumber \\&\quad - \sum \limits _{j} \int _{{\mathbb {R}}_+} \partial _t \alpha _2^{j}(t) \varphi \left( x_{j}+c_{j}(t-t_{j}),t \right) dt =0, \qquad \qquad \qquad \end{aligned}$$

where $\mathbf{U}^N = (U_1^N,U_2^N)$. Notice that, according to the construction (see Fig. 1), the interactions do not raise the BV-bound of the regular part of the solution, while the total sum of the strength-increment of the $\delta$-distributions can be bounded by the following expression (see (2.5)):

$$\begin{aligned} \max _{-M\le X,Y,{\tilde{X}},{\tilde{Y}}\le M}&\left\{ \left[ {\mathrm BV}(U_{01}) \, |\nabla {{\mathfrak {f}}}(X,Y)| + |f_1(X,Y)-f_1({\tilde{X}},{\tilde{Y}})| \, \right] \, T \right. \\&\left. + \left[ {\mathrm BV}(U_{02}) \, |\nabla {{\mathfrak {f}}}(X,Y)| + |f_2(X,Y)-f_2({\tilde{X}},{\tilde{Y}})| \, \right] \, T \right\} , \end{aligned}$$

where ${\mathrm BV}(w) = \sup \sum _{i}|w(x_i) - w(x_{i-1})|$ and *M* is the $L^{\infty }$ norm of the initial data. From here, we also conclude that $\partial _t \mathbf{U}^N$ is bounded in $L^1([0,T];W_{loc}^{-1,1}({\mathbb {R}}))$ since for any compactly supported $\varphi \in C^1_c([0,T]\times {\mathbb {R}})$

$$\begin{aligned} \Big | \int _{{\mathbb {R}}_+}\! \! \int _{{\mathbb {R}}}U_1^N \partial _t\varphi ~dxdt \Big | \le C \Vert \varphi \Vert _{L^\infty ([0,T]\times {\mathbb {R}})}. \end{aligned}$$

From here and since the BV-bound of the $L^\infty$-parts $U_1^N$ and $U_2^{N}$ of the families $u_1^{N}$ and $u_2^{N}$, respectively, are finite, according to the Aubin-Lions lemma, we conclude that $U_1^{N}$ and $U_2^{N}$ are strongly $L^1_{loc}$-precompact. Thus, they admit an $L^1_{loc}$ limit along a subsequence as $N \rightarrow \infty$, and we denote the limit by $U_1$ and $U_2$.

Moreover, since the sum of the strength of all $\delta$-functions from (2.16) remains uniformly bounded with respect to $\varepsilon$, for every $T>0$ there exists the weak limit to the functions from (2.16) in $Lip([0,T]\times \mathcal{M}_{loc}({\mathbb {R}}))$. In view of the previous paragraph, we denote these limits by

$$\begin{aligned}&u_1(t,x)= U_1(t,x)+m_1(t,x)\\&u_2(t,x)=U_2(t,x)+m_2(t,x). \end{aligned}$$

We can assume that the limit exists along the same subsequences of $(U_1^{N})$ and $(U_2^{N})$ as well as for the terms of the form (2.9).

Passing to the limit in (2.17) and (2.18) along the subsequence defining simultaneously the generalized tangential derivative of $m_1$ and $m_2$, and the converging subsequences of $(u_1^{N})$ and $(u_2^{N})$, we conclude that they satisfy (2.13). $\square$

## Weak Asymptotics

Close examination of the variational formulation (2.2) of weak singular solutions reveals that it is given in terms of the Rankine–Hugoniot deficit, and it is not entirely clear how this deficit is connected to a singular solution. Therefore, it is important to be able to construct the singular solutions as limits of approximate solutions of a regularized form of the equation. Following the method laid out in [17] and [30], we use the complex-valued weak asymptotic method in order to make the connection between the Rankine–Hugoniot deficit and the singular solutions containing $\delta$-distributions.

In order to outline the weak asymptotic method, let us first define a vanishing family of distributions.

### Definition 3.1

Let $f_{\varepsilon }(x) \in \mathcal{D}'({\mathbb {R}})$ be a family of distributions depending on $\varepsilon \in (0,1)$. We say that $f_{\varepsilon } = o_{\mathcal{D}'}(1)$ if for any test function $\phi (x)\in {\mathcal {D}}({\mathbb {R}})$, the estimate

$$\begin{aligned} \langle f_{\varepsilon },\phi \rangle =o(1), \ \ \mathrm{as}\ \ \varepsilon \rightarrow 0 \end{aligned}$$

holds.

The estimate on the right-hand side is understood in the usual Landau sense. Thus, we may say that a family of distributions approach zero in the sense defined above if for a given test function $\phi$, the pairing $\langle f_{\varepsilon },\phi \rangle$ converges to zero as $\varepsilon$ approaches zero.

### Definition 3.2

We say that the family of complex-valued distributions $(\mathbf{u}_\varepsilon )=(u_{1\varepsilon },\dots ,u_{d\varepsilon })$ represents a weak asymptotic solution to (1.1) if there exist real-valued distributions $\mathbf{u}=(u_{1},\dots ,u_{d})\in C({\mathbb {R}}_+;\mathcal{D}'({\mathbb {R}}))$, such that for every fixed $t\in {\mathbb {R}}_+=(0,\infty )$

$$\begin{aligned} \mathbf{u}_\varepsilon \rightharpoonup \mathbf{u}\ \ \mathrm{as} \ \ \varepsilon \rightarrow 0, \end{aligned}$$

in $$\mathcal{D}'({\mathbb {R}})$$, and

$$\begin{aligned}&\partial _t u_{1\varepsilon } + \partial _x f_1(\mathbf{u}_\varepsilon ) \ = \ {o}_{\mathcal{{D}'}}(1),\\&\qquad \dots \\&\partial _t u_{n\varepsilon } + \partial _x f_d(\mathbf{u}_\varepsilon ) \ = \ {o}_{\mathcal{{D}'}}(1). \end{aligned}$$

In the following, we will show that any $2\times 2$ system of conservation laws with polynomial flux function admits a sequence of approximate solutions converging towards a $\delta$-type solution of the Riemann problem. To this end, let us consider the system

3.1 $$\begin{aligned} \partial _t u+\partial _x \left( \sum \limits _{j=1}^{m}a_j u^{p_j} v^{q_j} \right)&=0 \end{aligned}$$

3.2 $$\begin{aligned} \partial _t v+\partial _x \left( \sum \limits _{j=1}^{n}b_j u^{r_j} v^{s_j} \right)&=0 \end{aligned}$$

where $a_1,\dots ,a_m$ and $b_1,\dots ,b_n$ are constants while $p_j, q_j$, $j=1,\dots ,m$, and $r_j, s_j$, $j=1,\dots ,n$, are non-negative integers. First, we shall formally extend the system by an equation which is trivially satisfied so that we can control our approximations. Without loss of generality, we assume that $s_n= \max \{s_1,\dots ,s_n\}>0$ and $b_n=1$, and we write

3.3 $$\begin{aligned}&\displaystyle \partial _t w =0 \end{aligned}$$

3.4 $$\begin{aligned}&\displaystyle \partial _t u+\partial _x \left( \sum \limits _{j=1}^{m}a_j w u^{p_j} v^{q_j} \right) =0 \end{aligned}$$

3.5 $$\begin{aligned}&\displaystyle \partial _t v+\partial _x \left( u^{r_n}v^{s_n} +\sum \limits _{j=1}^{n-1}b_j w u^{r_j} v^{s_j} \right) =0 \end{aligned}$$

with the condition $w(0,x)=1$ and, for simplicity, $r_n\ne 0$. The case $r_n=0$ can be handled similarly. Clearly, $w\equiv 1$ and the systems (3.1), (3.2), and (3.3), (3.4), (3.5) are equivalent. But since we need to control approximations on infinitesimal intervals, the function *w* (or more precisely its approximation) will play a substantial role. We have the following theorem:

### Theorem 3.1

Assume that $w|_{t=0}(x)=1$ and

$$\begin{aligned} u|_{t=0}(x)={\left\{ \begin{array}{ll} u_L, &{} x<0\\ u_R, &{} x \ge 0 \end{array}\right. }, \ \ v|_{t=0}(x)={\left\{ \begin{array}{ll} v_L, &{} x<0\\ v_R, &{} x \ge 0 \end{array}\right. }. \end{aligned}$$

Then, there exist (complex valued) families $(u_\varepsilon )$, $(v_\varepsilon )$ and $(w_\varepsilon )$ such that

3.6 $$\begin{aligned}&\displaystyle \partial _t w_{\varepsilon } =o_{\mathcal{D}'}(1), \end{aligned}$$

3.7 $$\begin{aligned}&\displaystyle \partial _t u_\varepsilon +\partial _x \left( \sum \limits _{j=1}^{m}a_j w_{\varepsilon } u_\varepsilon ^{p_j} v_\varepsilon ^{q_j} \right) =o_{\mathcal{D}'}(1), \end{aligned}$$

3.8 $$\begin{aligned}&\displaystyle \partial _t v_\varepsilon +\partial _x \left( u_{\varepsilon }^{r_n}v_{\varepsilon }^{s_n} + \sum \limits _{j=1}^{n-1}b_j w_{\varepsilon } u_\varepsilon ^{r_j} v_\varepsilon ^{s_j} \right) =o_{\mathcal{D}'}(1), \end{aligned}$$

such that

$$\begin{aligned}&w_{\varepsilon }\big |_{t=0} \rightharpoonup 1 \ \ \mathrm{in} \ \ \mathcal{D}'({\mathbb {R}}), \ \ \mathrm{and} \ \ w_{\varepsilon } \rightharpoonup 1 \ \ \mathrm{in} \ \ \mathcal{D}'({\mathbb {R}}_+\times {\mathbb {R}}),\\&u_\varepsilon \big |_{t=0} \rightharpoonup {\left\{ \begin{array}{ll} u_L, &{} x<0\\ u_R, &{} x \ge 0 \end{array}\right. } \ \ \ \ \mathrm{in} \ \ \mathcal{D}'({\mathbb {R}}), \\&v_\varepsilon \big |_{t=0} \rightharpoonup {\left\{ \begin{array}{ll} v_L, &{} x<0\\ v_R, &{} x \ge 0 \end{array}\right. } \ \ \mathrm{in} \ \ \mathcal{D}'({\mathbb {R}}). \end{aligned}$$

Moreover,where *c* is given by the Rankine–Hugoniot conditions from Eq. (3.1)while $\alpha$ is the Rankine–Hugoniot deficit.

### Proof

Fix large $M>0$ (say $M>100$) and consider the following approximation $w_{\varepsilon }$:

$$\begin{aligned} w_\varepsilon (t,x)={\left\{ \begin{array}{ll} 1, &{} x\notin (ct-M\varepsilon ,ct+M\varepsilon )\\ 0, &{} x\in [ct-M\varepsilon ,ct+M\varepsilon ] \end{array}\right. }. \end{aligned}$$

Then, let

$$\begin{aligned} u_\varepsilon (t,x)={\left\{ \begin{array}{ll} u_L, &{} x<ct -M \varepsilon \\ 1, &{} x\in [ct-M \varepsilon ,ct)\\ 0, &{} x\in [ct,ct+M\varepsilon ]\\ u_R, &{} x>ct+M\epsilon \end{array}\right. }. \end{aligned}$$

From the above assumptions, we see that (3.7) reduces to

3.9 $$\begin{aligned}&\partial _{t}u_\varepsilon + \partial _{x} \left( \sum \limits _{j=1}^{m}a_j u_L^{p_j} v_L^{q_j} \chi _{\{-\infty ,-M\varepsilon \}}(x-ct)\right. \nonumber \\&\left. + \sum \limits _{j=1}^{m}a_j u_R^{p_j} v_R^{q_j} \chi _{\{M\varepsilon ,\infty \}}(x-ct) \right) =o_{\mathcal{D}'}(1), \end{aligned}$$

and this is clearly satisfied according to the choice of the speed *c*.

As for the family $(v_\varepsilon )$, we take a standard approximation of the Dirac distribution: $(\delta _\varepsilon )$ is non-negative family of functions compactly supported at $(-M\varepsilon ,0)$

$$\begin{aligned} \delta _\varepsilon (x) \rightharpoonup \delta (x), \ \ \mathrm{supp}\delta _{\varepsilon } \subset (-M\varepsilon ,0). \end{aligned}$$

Then we take

3.10 $$\begin{aligned} v_\varepsilon (t,x)={\left\{ \begin{array}{ll} v_L, &{} x<ct -M_\varepsilon ,\\ \root s_n \of {c\alpha (t)\delta _\varepsilon (x-ct)}, &{} x\in [ct-M\varepsilon ,ct),\\ \delta _\varepsilon (x-ct-M\varepsilon ), &{} x\in (ct,ct+M\varepsilon ],\\ u_R, &{} x>ct+M\epsilon . \end{array}\right. } \end{aligned}$$

Observe that the functions $(v_\varepsilon )$ may not be real-valued. If we notice that

$$\begin{aligned} \root s_n \of {c\alpha (t)\delta _\varepsilon (x-ct)} \rightarrow 0 \ \ \mathrm{in} \ \ \mathcal{D}'({\mathbb {R}}_+\times {\mathbb {R}}), \end{aligned}$$

Eq. (3.5) becomes in the limit

$$\begin{aligned} \left( -c(v_R-v_L)+\alpha '(t) \right) \delta (x-ct) + \left( \sum \limits _{j=1}^{n}a_j u_R^{s_j} v_R^{r_j}-\sum \limits _{j=1}^{n}a_j u_L^{s_j} v_L^{r_j} \right) \delta (x-ct)=0. \end{aligned}$$

Thus, choosing $\alpha '(t)$ to be the Rankine–Hugoniot deficit, we conclude the theorem. $\square$

We note that by introducing more auxiliary equations of the type $\partial _t w=0$, $w(0,x)=1$ and positioning them in appropriate places in the original equation, we can get a large number of different approximate solutions consistent with the conclusions of Theorem 2.1.

## Discussion

One natural question to ask is whether the appearance of $\delta$-type solutions is physically reasonable. Many of the systems which were shown to have these singular solutions were found using mathematical rather than physical considerations. Nevertheless, it may be argued that $\delta$-type solutions appear rather naturally especially from a modeling point of view.

First of all, $\delta$-distributions are experimentally confirmed as solutions of systems of conservation laws. This has recently been observed in [44] in nonlinear chromatography. Namely, during an experiment involving chemical interactions of different substances, the authors of [44] noticed abrupt increment of concentrations on specific isolated sets. The results are confirmed mathematically in several papers where the system of chromatography equations is considered (cf. [36, 62, 65]). Similar concentration effects were also observed in a system modeling the polymer flooding of a porous medium [26], in pressureles gas dynamics [67] and in thin-film equations [40, 59].

The necessity to consider Rankine–Hugoniot deficits and corresponding singular solutions can also be understood in terms of conservation of mass, momentum and energy. Consider the the shallow-water system

4.1 $$\begin{aligned} \partial _t h + \partial _x \left( u h \right)&= \ 0, \end{aligned}$$

4.2 $$\begin{aligned} \partial _t u + \partial _x \left( h + { \textstyle \frac{u^2}{2}} \right)&= \ 0. \end{aligned}$$

If the solutions of the system are smooth, they also satisfy a corresponding conservation equation for horizontal momentum given by

4.3 $$\begin{aligned} \begin{array}{ll} \partial _t( uh) + \partial _x \left( hu^2 + \frac{1}{2} h^2 \right) &{} 0 , \\ \end{array} \end{aligned}$$

and an energy equation which takes the form

4.4 $$\begin{aligned} \begin{array}{ll} \partial _t\left( \frac{1}{2} hu^2 + \frac{1}{2} h^2 \right) + \partial _x \left( \frac{1}{2} hu^3 + u h^2 \right)&0. \end{array} \end{aligned}$$

**Fig. 2:**
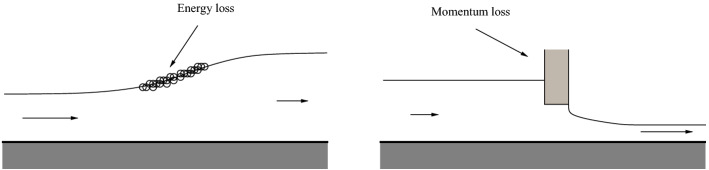
Left panel: schematic picture of a hydraulic jump. Momentum is conserved, but energy is lost due to surface undulations and turbulence. Right panel: flow and under a sluice gate. Momentum loss occurs, but energy is conserved

Let us consider the situations sketched in Fig. 2. In both cases given there, the solutions develop discontinuities. Since mass should always be conserved, we need to decide whether to pair mass conservation with momentum or energy conservation in order to find appropriate solutions.

The phenomenon occurring in the case shown in the left panel is called a hydraulic jump which is frequently observed in open channel flow such as rivers and spillways. In this case, it is well known that the conservation of mass and momentum is required [25]. If the hydraulic jump is traveling at a speed *c*, the corresponding Rankine–Hugoniot conditions take the form

$$\begin{aligned} c [h] =&[ hu],\\ c [ hu] =&\left[ hu^2 + { \textstyle \frac{1}{2}} h^2 \right] . \end{aligned}$$

It is also well known that there is a loss of energy across the transition region [2, 66], even if the flow is not turbulent.

On the other hand, in the flow under a sluice gate such as shown in the right panel of Fig. 2, mass and energy are conserved. Momentum is lost because the sluice gate exerts a force *F* on the fluid. However energy is conserved since when the fluid touches the gate, its velocity is zero. Therefore, no work is done ($dW/dt = F u$ where *W* is the energy, and $u = 0$). Thus the Rankine–Hugoniot conditions take the form

$$\begin{aligned} c [h] =&[ hu],\\ c \left[ { \textstyle \frac{1}{2}}hu^2 + { \textstyle \frac{1}{2}}h^2 \right] =&\left[ { \textstyle \frac{1}{2}} hu^3 + u h^2 \right] , \end{aligned}$$

where most appropriately, $c=0$.

Let us now assume that we have both situations simultaneously, i.e. a flow containing both a traveling hydraulic jump and a passage under a sluice gate in an adjacent section of the channel. If both phenomena are to be described by a single system of two equations (since they happen simultaneously), a natural choice is the system (4.1) and (4.2). Locally, proper discontinuous solutions are obtained using the Rankine–Hugoniot conditions corresponding to either equations (4.1) and (4.3) for the hydraulic jump, or to (4.1) and (4.4) for the flow under the sluice gate. These local solutions will not in general satisfy the Rankine–Hugoniot conditions corresponding to (4.1) and (4.2), and it can be shown in particular in the case of a traveling hydraulic jump that a nonzero Rankine–Hugoniot deficit must occur in (4.2). However, this deficit can be handled by incorporating a $\delta$-distribution into the solution *u*.

The necessity to go beyond the standard BV-solutions can also be ascribed to the use of mathematically ineligible operations during the model derivation; for instance the tacit assumption on smoothness of solutions. Indeed, it is well understood that the introduction of the notion of weak solution causes non-uniqueness. In the case of scalar conservation laws, this imperfection is recovered using the Kruzhkov entropy concept [38] but only if the flux is Lipschitz continuous. Clearly, the number of possibly ineligible operations is greater in the case of systems of conservation laws, and may cause a variety of problems such as non-existence and non-uniqueness.

Indeed, uniqueness is an issue which is quite rarely considered in the framework of $\delta$-shock solutions. Uniqueness was obtained in [28] using entropy inequalities of Oleinik-type [49], and in [47] with the help of entropy inequalities of Kruzhkov-type [38]. These methods may yield results in special cases, but they do not offer a clear reason why the uniqueness is gained, and what the entropy inequality means physically or even mathematically.

The weak asymptotic method appears to lead to the correct choice of the shock speed for singular solutions. In fact, in cases where the equations have singular solutions given in exact form, the weak asymptotic method can be shown to give the right solution (cf. [34, 56] for example). However, there is not a firm proof that the weak asymptotic method works in every case.

It is our belief that one of the reasons for the lack of a rigorous uniqueness concept, beside obvious mathematical difficulties, essentially lies in the oversimplification of the physical system, leading to ambiguities in the mathematical treatment. One possible remedy would be to consider the shock structure as part of the system, such as for example explained in [66]. However, then the relative simplicity of the conservation laws would also be lost.

## Data Availability

Data sharing is not applicable to this article as no new data were created or analyzed in this study.

## Footnotes

1 By genuinely nonlinear nonlinear, we mean that each wave family is either genuinely nonlinear or linearly degenerate.

2 The space *BV*(*I*) is defined as the set of functions *u*(*x*) for which $\sup \sum _{i}|u(x_i) - u(x_{i-1})|$ is finite, where the sum is taken over all finite partitions $\{x_i\}$ of the interval *I* with $x_{i-1} < x_i$
